# Influence of temperature and light on total phenolic compounds during natural orange juice storage

**DOI:** 10.1080/07853890.2021.1896082

**Published:** 2021-09-28

**Authors:** C. Capitão, D. Coutinho, B. Dias, R. Ramalho, P. Pereira

**Affiliations:** a Instituto Universitário Egas Moniz (IUEM), Egas Moniz Cooperativa de Ensino Superior, Caparica, Portugal; b Centro de Investigação Interdisciplinar Egas Moniz (CiiEM), Egas Moniz Cooperativa de Ensino Superior, Caparica, Portugal; c Grupo de Estudos em Nutrição Aplicada (GENA), Instituto Universitário Egas Moniz (IUEM), Egas Moniz Cooperativa de Ensino Superior, Caparica, Portugal

## Abstract

**Introduction:**

Natural orange juice is a phenolic compounds rich beverage, whose disposal on market and food catering has increased with the expansion of the concept of healthy food, raising concerns about the effect of storage on total antioxidant capacity (TAC), and scientific evidence on this is scarce. Regarding TAC, is well established that phenolic compounds account more to it than ascorbic acid [1]. Our aim was to evaluate the concentration of total phenolic compounds (CTPC) in fresh squeezed orange juice and analyse the influence of storage conditions (temperature and light) during 48 h on CTPC.

**Materials and methods:**

Fresh oranges (*Citrus sinensis,* variety “Valencia Late”, Portugal) of the same calibre and producer were squeezed aliquoted into 3 glasses stored in different conditions: (1) Room temperature (20 °C) and exposed to sunlight; (2) Room temperature (20 °C) fully wrapped in aluminium; and (3) Refrigerated (1 °C). The procedure was performed according to the methodology described by Keskin-Šašić et al. [2]. CTPC was quantified by Folin-Ciocalteu spectrophotometric method, using gallic acid as standard, at 0, 2.5, 4, 8 and 48 h. (a) Samples preparation: 1 mL of orange juice was diluted from each vial in water, to a volume of 25 mL. Part of solution was centrifuged at 5300 rpm for 20’. The supernatant solution was used to analyse. (b) Determination of CTPC: approximately 0.2 mL of each sample was transferred to test tubes containing 1.0 mL of a dilution of Folin–Ciocalteu reagent in water (1:10). After 10’, 0.8 mL of a sodium carbonate solution (7.5% m/v) was added to the sample. The test tubes were allowed to stand at room temperature for 30’, and then absorbance was measure at 743 nm. CTPC was expressed as equivalents of gallic acid (EGA) in mg/100 ml of squeezed orange juice. Samples’ CTPC was determined from a standard curve of gallic acid varying between 0.2 and 4 mg/L.

**Results:**

CTPC of recently squeezed fresh oranges was 30.1 mg EGA/100 mL (basal). After 48 h, CTPC decreased in all samples: 38.5% in sample 1 (18.5 mg EGA/100 mL), 28.6% in sample 2 (21.8 mg EGA/100 mL), and 27.6% in sample 3 (21.5 mg EGA/100 mL). The higher variation in CTPC occurred for sample 1, stored at room temperature and exposed to sunlight. **Discussion and conclusions:** Our results suggest that exposure to sunlight is the variable that most influences the decrease CTPC of the fresh orange juice after 48 h. More studies on the influence of these variables are needed to confirm these results and to increase knowledge about storage of fruit fresh juices, to ensure the nutritional quality of this product. Squeezed orange juice is widely present on markets and coffee shops, usually refrigerated but not protected from sunlight, which means that the storage conditions would need to be reconsidered.
